# Depression, anxiety and associated factors among Chinese adolescents during the COVID-19 outbreak: a comparison of two cross-sectional studies

**DOI:** 10.1038/s41398-021-01271-4

**Published:** 2021-03-02

**Authors:** Xu Chen, Han Qi, Rui Liu, Yuan Feng, Wen Li, Mi Xiang, Teris Cheung, Todd Jackson, Gang Wang, Yu-Tao Xiang

**Affiliations:** 1 The National Clinical Research Center for Mental Disorders & Beijing Key Laboratory of Mental Disorders, Beijing Anding Hospital & the Advanced Innovation Center for Human Brain Protection, Capital Medical University, Beijing, China; 2 Unit of Psychiatry, Institute of Translational Medicine, Faculty of Health Sciences, University of Macau, Macao SAR, China; 3 Center for Cognition and Brain Sciences, University of Macau, Macao SAR, China; 4 Institute of Advanced Studies in Humanities and Social Sciences, University of Macau, Macao SAR, China; 5 School of Public Health, Shanghai Jiao Tong University, Shanghai, China; 6 School of Nursing, Hong Kong Polytechnic University, Hong Kong SAR, China; 7 Department of Psychology, University of Macau, Macao SAR, China

**Keywords:** Depression, Psychiatric disorders

## Abstract

The 2019 coronavirus disease (COVID-19) is a public health emergency of international concern. In China, all schools were shut down and students were home quarantined to prevent disease spread; these steps could have potential negative effects on mental health of adolescents. This study aimed to examine changes in depression and anxiety among Chinese adolescents during the COVID-19 epidemic, and explore factors associated with depression and anxiety. Two survey administrations were conducted among Chinese adolescents between February 20 and February 27 and between April 11 and April 19, 2020, respectively. The Center for Epidemiological Studies-Depression Scale (CES-D) and the 7-item Generalized Anxiety Disorder (GAD-7) scale were used to assess depressive symptoms and anxiety symptoms, respectively. A total of 9554 and 3886 adolescents participated in the first and second surveys. During the initial survey, the prevalence of depression was 36.6% (95% CI: 35.6–37.6%) while the prevalence of anxiety was 19% (95% CI: 18.2–19.8%). Rates of depression and anxiety increased to 57.0% (95% CI: 55.4–58.6%) and 36.7% (95% CI: 35.2–38.2%), respectively, in the second survey. Multivariable logistic regression analyses revealed that group membership in the second survey, female gender, senior secondary school enrollment, and concerns about entering a higher grade were positively associated with both depression and anxiety. Conversely, a sleep duration of ≥6 h/day, an exercise duration ≥30 min/day, having the same as typical or higher study efficiency during the COVID-19 outbreak, and living in provinces with 1000–9999 confirmed COVID-19 cases were negatively associated with depression and anxiety. In conclusion, compared to figures reported during the COVID-19 outbreak, the prevalence of depression and anxiety in Chinese adolescents significantly increased after the initial outbreak. Regular screening and appropriate interventions are urgently needed to reduce the risk for emotional disturbances among adolescents during and after the initial COVID-19 outbreaks.

## Introduction

Coronavirus disease 2019 (COVID-19) was first reported in December 2019 in Wuhan, Hubei province, China^1^ and was subsequently reported in more than 200 countries and territories^2^. On January 30, 2020, the World Health Organization (WHO) declared COVID-19 to be a public health emergency of international concern^3^. Due to fear of infection, potentially fatal consequences of COVID-19 and related problems such as unemployment, financial strain, quarantine and long-term lockdown in some areas, mental health problems resulting from the COVID-19 outbreak are common in different subpopulations including confirmed patients^4^, frontline health professionals, and the elderly^5–7^. However, to date very few studies have focused on mental health of adolescents during the pandemic, although this subpopulation is also vulnerable to experiencing mental health problems^8,9^.

To reduce rapid transmission, the Chinese Ministry of Education suggested postponement of the spring 2020 semester in primary and secondary schools on January 30, 2020. Consequently, all face-to face teaching in schools has been suspended in China during the COVID-19 pandemic and students must receive education online at home^10,11^. As such, primary and secondary school students in China have stayed at home and have had to communicate with their classmates and teachers via the internet or other social media forums for more than 5 months. Although some schools in China were reopened after the initial COVID-19 outbreak was well controlled in April, 2020^12^, many schools remain closed due to the fear of a second outbreak. Prolonged home stay is associated with a range of negative outcomes for adolescents, such as decreased social interaction with peers, reduced physical activity, increased conflicts with parents, and academic pressures due to sudden changes of traditional learning methods^13–15^. Presumably, all of these changes could increase risk for mental health problems, particularly depression and anxiety. For example, a recent cross-sectional study has found the prevalence of depression and anxiety to be 43.7% and 37.4%, respectively, among Chinese adolescents during the COVID-19 outbreak^16^. However, to date, comparisons of the mental health status of adolescents have not been made between different stages of the COVID-19 outbreak. Thus, we set out to compare the prevalence of depression and anxiety among adolescents at different stage of COVID-19 outbreak as well as correlates of these experiences. Considering that the impact of persistent financial crisis on parental behaviors^8,17^, potential intensification of social isolation and loneliness^18^, and cumulative pressure of long-term online learning due to the COVID-19 outbreak could increase the risk of mental health problems among adolescents, we hypothesized that the prevalence of depression and anxiety would increase among Chinese adolescents over time.

## Methods

### Settings and participants

In this national study, two administrations of a mental health survey were conducted through the collaborative research network of the National Clinical Research Center for Mental Disorders, China; all 34 provinces of China were included. The first survey was administered from February 20 to 27 (during the COVID-19 outbreak) while the second round was administered from April 11 to 19 (after the COVID-19 outbreak). Due to risk of infection, traditional face-to-face interviews were not adopted. Following other studies^19–21^, snowball sampling was used, and data were collected using a smart phone-based WeChat-Wenjuanxing application. The application has more than 1 billion users in China and has been widely used for student management in most secondary schools in China during the COVID-19 outbreak. Inclusion criteria included: (1) secondary school students aged between 11 and 20 years; and (2) living in China during the COVID-19 outbreak. Participants with pre-existing major psychiatric disorders (e.g., psychotic disorder, major depression, and autism) were excluded based on participant self-reports. All participants were required to provide written informed consent. For participants under 18 years old, guardians were required to provide written consent. This study was approved by the Medical Ethical Committee of Beijing Anding Hospital, Capital Medical University, China.

### Data Collection

A pre-designed data collection form was used to collect socio-demographic data and information about clinical characteristics. Study sites were classified according to the total number of COVID-19 patients at provincial level based on a report of the National Health Commission of China (http://www.nhc.gov.cn) released on February 27, 2020.

Depression was assessed with the Chinese version of the Center for Epidemiological Studies-Depression Scale (CES-D)^22^. The CES-D is a 20-item self-report questionnaire^23^. Each item scored from 0 (not at all) to 3 (a lot). CES-D total scores of >15 were considered “having depression”^24^. The Chinese version of CES-D has been validated in adolescents with satisfactory psychometric properties^25^. Anxiety symptoms were measured using the 7-item self-report Generalized Anxiety Disorder Scale (GAD-7). Each item scored from 0 (not at all) to 3 (nearly every day)^26^, with total scores ranging from 0 to 21. Cutoff values of 5, 10, 15 indicate mild, moderate, and severe anxiety, respectively^27^. In this study, participants who scored >4 on the GAD-7 were classified as having anxiety. The Chinese version of GAD-7 has satisfactory psychometric properties^28^.

### Statistical analysis

All analyses were performed with SPSS version 21.0 software. A subset of adolescents who completed the first survey administration also participated in the second administration. Therefore, to avoid any overlap between the two study samples, those who participated in both surveys were excluded from the second sample. Mann–Whitney *U*-tests were used to compare CES-D and GAD-7 total scores between the two surveys. Demographic correlates of depression and anxiety during each assessment were assessed using chi-square tests. Multivariable logistic regression analyses with “enter” method were performed to examine correlates of depression and anxiety, respectively. In these analyses, either depression or anxiety was entered as the dependent variable while significant correlates based on univariate analyses were independent variables. A *P* value of <0.05 level was considered statistically significant (two-tailed).

## Results

### Demographic characteristics for the first and second survey

Of 9744 adolescents who enrolled in the initial survey, 9554 (47.9% male) completed the assessment and their responses were included in analyses. In addition, of 10,706 adolescents who volunteered for the second survey administration, 10,605 (46.2% male) completed the assessment. After excluding students who had also completed the initial survey (*n* = 6719), the final sample included for analyses from the second survey comprised 3886 students. Using power analysis^29^, the achieved power of the two surveys in this study was greater than 0.9, indicating that the sample size was large enough. Demographic characteristics based on presence/absence of depression and anxiety were summarized in Table 1.

**Table 1:** The comparison of CES-D and GAD-7 scores between the first (*n* = 9554) and second (*n* = 3886) surveys.

Variables	Scores of CES-D				Scores of GAD-7			
	The first survey	The second survey	*Z*	*P value*	The first survey	The second survey	*Z*	*P value*
Gender								
male	13.17 ± 10.13	18.74 ± 11.66	−18.09	**<0.001**	1.98 ± 3.37	3.77 ± 4.49	−17.47	**<0.001**
female	14.87 ± 11.33	19.75 ± 11.93	−16.79	**<0.001**	2.55 ± 3.66	4.16 ± 4.43	−17.43	**<0.001**
Age (years)								
11–15	12.67 ± 10.27	17.77 ± 11.96	−15.43	**<0.001**	1.95 ± 3.28	3.58 ± 4.52	−14.12	**<0.001**
16–20	16.03 ± 11.23	20.18 ± 11.65	−14.50	**<0.001**	2.75 ± 3.83	4.22 ± 4.41	−16.22	**<0.001**
Grade								
Junior secondary school	12.66 ± 10.31	17.69 ± 12.12	−14.25	**<0.001**	1.94 ± 3.29	3.56 ± 4.62	−12.62	**<0.001**
Senior secondary school	15.91 ± 11.16	20.06 ± 11.60	−14.95	**<0.001**	2.73 ± 3.79	4.19 ± 4.37	−16.60	**<0.001**
School type								
Provincial key schools	13.95 ± 10.87	19.99 ± 11.97	−7.12	**<0.001**	2.64 ± 4.09	4.77 ± 4.46	−8.78	**<0.001**
Municipal key schools	13.01 ± 10.58	19.08 ± 12.67	−9.06	**<0.001**	2.37 ± 3.64	4.28 ± 4.99	−8.35	**<0.001**
Ordinary schools	13.75 ± 10.69	19.52 ± 11.77	−22.90	**<0.001**	2.16 ± 3.43	3.97 ± 4.41	−22.33	**<0.001**
Vocational schools	15.86 ± 11.09	16.10 ± 10.40	−0.77	0.441	2.43 ± 3.53	2.86 ± 4.02	−1.18	0.237
Major class	13.12 ± 10.70	20.55 ± 11.52	−18.20	**<0.001**	2.38 ± 3.70	4.27 ± 4.32	−15.92	**<0.001**
Sleep duration/day								
<6 h	22.06 ± 13.50	26.30 ± 13.28	−4.79	**<0.001**	4.94 ± 5.65	6.67 ± 6.01	−4.95	**<0.001**
6–8 h	14.56 ± 10.70	19.11 ± 11.32	−18.30	**<0.001**	2.39 ± 3.45	3.84 ± 4.13	−18.52	**<0.001**
>8 h	12.38 ± 10.06	15.37 ± 10.89	−6.89	**0.019**	1.81 ± 3.15	2.83 ± 3.90	−6.90	**<0.001**
Study duration/day								
<4 h	16.66 ± 11.49	17.97 ± 11.57	−2.06	**0.039**	2.68 ± 4.00	3.63 ± 4.54	−3.80	**<0.001**
4–8 h	13.30 ± 10.19	18.46 ± 11.38	−19.29	**<0.001**	2.08 ± 3.24	3.64 ± 4.17	−18.57	**<0.001**
>8 h	13.98 ± 11.22	21.53 ± 12.50	−17.84	**<0.001**	2.40 ± 3.72	4.80 ± 4.87	−17.13	**<0.001**
Exercise duration/day								
<30 min	16.13 ± 11.31	21.65 ± 12.08	−17.22	**<0.001**	2.68 ± 3.82	4.55 ± 4.67	−17.82	**<0.001**
30–60 min	12.29 ± 9.90	16.89 ± 10.73	−15.89	**<0.001**	1.91 ± 3.15	3.35 ± 3.97	−15.52	**<0.001**
>60 min	12.31 ± 10.57	17.82 ± 12.55	−7.93	**<0.001**	2.10 ± 3.62	3.87 ± 4.88	−7.42	**<0.001**
Change of study efficiency								
Lower than before	17.25 ± 11.63	20.67 ± 11.94	−11.38	**<0.001**	3.03 ± 4.10	4.33 ± 4.53	−13.68	**<0.001**
About the same	12.60 ± 9.97	16.40 ± 10.98	−11.14	**<0.001**	1.87 ± 3.10	3.20 ± 4.09	−10.73	**<0.001**
Higher than before	12.18 ± 10.39	17.26 ± 11.62	−6.32	**<0.001**	2.19 ± 3.53	3.70 ± 4.82	−4.86	**<0.001**
No. of COVID-19 cases at provincial level								
10–99	14.07 ± 11.16	24.17 ± 13.54	−1.19	0.235	2.70 ± 4.03	2.50 ± 3.27	−0.77	0.442
100–999	14.29 ± 10.61	19.49 ± 11.86	−22.89	**<0.001**	2.22 ± 3.38	4.08 ± 4.50	−23.23	**<0.001**
1000–9999	13.08 ± 10.86	17.39 ± 11.63	−5.74	**<0.001**	1.94 ± 3.32	2.83 ± 3.97	−3.91	**<0.001**
>10,000	14.59 ± 11.17	16.63 ± 10.28	−2.46	**0.014**	2.41 ± 3.66	3.16 ± 3.51	−2.75	**0.006**
Participating in distance learning	13.80 ± 10.73	19.29 ± 11.81	−25.57	**<0.001**	2.24 ± 3.52	3.97 ± 4.44	−24.96	**<0.001**
Concerned about entering a higher grade	16.32 ± 11.29	20.70 ± 11.80	−16.16	**<0.001**	2.83 ± 3.95	4.39 ± 4.55	−17.64	**<0.001**

Bolded Values: *P* < 0.05.

The scores of CES-D and GAD-7 were presented as mean ± standard deviation.

*CES-D* Center for Epidemiological Studies-Depression Scale, *GAD-7* 7-item Generalized Anxiety Disorder Scale.

### Depression and anxiety in the first and second survey

Mean CES-D and GAD-7 total scores were compared between the first and second surveys in Table 1. The mean CES-D total score for the whole sample was 14.06 ± 10.80 in the first survey and 19.29 ± 11.82 in the second survey (*Z* = −24.78, *P* < 0.001). Mean GAD-7 total score also increased from 2.28 ± 3.54 in the first survey to 3.98 ± 4.46 in the second survey (*Z* = −24.88, *P* < 0.001). Except for students in vocational schools and those living in provinces with 10–99 confirmed COVID-19 cases, mean CES-D and GAD-7 total scores based on demographic characteristics and study-related variables were also significantly higher in the second survey than the first survey (Table 1).

Prevalence estimates of depression (CES-D > 15) and anxiety (GAD-7 > 4) were also compared between the surveys. In the first survey, the prevalence of depression was 36.6% (95% CI: 35.6–37.6%) while the rate of anxiety was 19% (95% CI: 18.2–19.8%). In the second survey, corresponding figures were 57.0% (95% CI: 55.4–58.6%) for depression and 36.7% (95% CI: 35.2–38.2%) for anxiety. Table 2 breaks down depression and anxiety prevalence stratified by demographic characteristics and study-related variables.

**Table 2:** The comparison of the prevalence of depression and anxiety between the first and second surveys.

Variables	Depression (CES-D > 15) *n* = 5713				Anxiety (GAD-7 > 4) *n* = 3240			
	The first survey	The second survey	*X*^*2*^	*P value*	The first survey	The second survey	*X*^*2*^	*P value*
Gender								
male	1514 (33.1)	962 (54.8)	252.31	**<0.001**	759 (16.6)	614 (35.0)	253.41	**<0.001**
female	1984 (39.9)	1253 (58.8)	215.16	**<0.001**	1,055 (21.2)	812 (38.1)	219.85	**<0.001**
Age (years)								
11–15	1745 (31.1)	721 (50.6)	188.68	**<0.001**	872 (15.6)	465 (32.6)	214.82	**<0.001**
16–20	1753 (44.4)	1494 (60.7)	161.45	**<0.001**	942 (23.9)	961 (39.1)	167.66	**<0.001**
Grade								
Junior secondary school	1693 (31.0)	617 (48.9)	145.24	**<0.001**	848 (15.5)	414 (32.8)	200.48	**<0.001**
Senior secondary school	1805 (44.1)	1598 (60.9)	181.04	**<0.001**	966 (23.6)	1,012 (38.6)	172.70	**<0.001**
School type								
Provincial key schools	223 (36.9)	158 (58.7)	36.01	**<0.001**	138 (22.8)	124 (46.1)	47.89	**<0.001**
Municipal key schools	537 (32.4)	204 (55.4)	68.68	**<0.001**	319 (19.3)	133 (36.1)	49.45	**<0.001**
Ordinary schools	1891 (35.1)	1742 (58.1)	413.57	**<0.001**	947 (17.6)	1103 (36.8)	383.93	**<0.001**
Vocational schools	847 (44.4)	111 (44.6)	0.003	0.956	410 (21.5)	66 (26.5)	3.224	0.073
Major class	799 (32.7)	657 (63.7)	285.62	**<0.001**	493 (20.2)	417 (40.4)	153.56	**<0.001**
Sleep duration/day								
<6 h	283 (63.5)	322 (75.9)	16.01	**<0.001**	185 (41.5)	233 (55.0)	15.81	**<0.001**
6–8 h	2089 (39.0)	1626 (57.3)	252.07	**<0.001**	1085 (20.2)	1029 (36.3)	249.18	**<0.001**
>8 h	1126 (30.0)	267 (42.7)	39.35	**<0.001**	544 (14.5)	164 (26.2)	54.02	**<0.001**
Study duration/day								
<4 h	704 (45.7)	163 (50.9)	2.97	0.085	341 (22.1)	105 (32.8)	16.65	**<0.001**
4–8 h	1711 (34.3)	1298 (54.4)	270.35	**<0.001**	862 (17.3)	796 (33.3)	239.77	**<0.001**
>8 h	1083 (35.9)	695 (64.8)	269.47	**<0.001**	611 (20.3)	479 (44.7)	241.47	**<0.001**
Exercise duration/day								
<30 min	1924 (43.8)	1.232 (65.5)	247.84	**<0.001**	999 (22.7)	799 (42.5)	250.76	**<0.001**
30–60 min	1289 (30.3)	777 (48.9)	174.43	**<0.001**	651 (15.3)	492 (31.0)	179.82	**<0.001**
>60 min	285 (31.3)	206 (49.5)	40.92	**<0.001**	164 (18.0)	135 (32.5)	34.29	**<0.001**
Change of study efficiency								
Lower than before	1449 (47.1)	1605 (61.8)	122.36	**<0.001**	791 (25.7)	1032 (39.7)	126.97	**<0.001**
About the same	1751 (31.7)	514 (47.0)	94.83	**<0.001**	849 (15.4)	332 (30.4)	139.76	**<0.001**
Higher than before	298 (31.1)	96 (49.0)	23.21	**<0.001**	174 (18.1)	62 (31.6)	18.21	**<0.001**
No. of COVID-19 cases at provincial level								
10–99	21 (58.3)	4 (66.7)	0.15	0.700	17 (47.2)	2 (33.3)	0.40	0.527
100–999	2721 (37.2)	2067 (58.2)	424.92	**0.002**	1415 (19.4)	1332 (37.5)	415.41	**<0.001**
1000–9999	560 (33.1)	91 (44.2)	9.92	**<0.001**	277 (16.4)	53 (25.7)	11.14	**0.001**
>10,000	196 (37.5)	53 (43.8)	1.62	0.203	105 (20.1)	39 (32.2)	8.30	**0.004**
Participating in distance learning	3181 (35.5)	2156 (57.0)	504.96	**<0.001**	1672 (18.7)	1380 (36.5)	463.79	**<0.001**
Concerned about entering a higher grade	1955 (44.9)	1789 (62.4)	213.09	**<0.001**	1052 (24.1)	1183 (41.3)	237.32	**<0.001**

Bolded Values: *P* < 0.05.

The percentages of CES-D > 15 and GAD-7 > 4 were presented as *n* (%).

*CES-D* Center for Epidemiological Studies-Depression Scale, *GAD-7* 7-item Generalized Anxiety Disorder Scale.

### Correlates of depression and anxiety

Multivariable logistic regression analyses revealed that increased risk of depression was associated with completing the second survey (OR = 1.47, 95% CI: 1.34–1.61), female gender (OR = 1.21, 95% CI: 1.12–1.31), senior secondary school (OR = 1.41, 95% CI: 1.29–1.54), vocational schools (OR = 1.25, 95% CI: 1.04–1.51), and being concerned about entering a higher grade (OR = 1.75, 95% CI: 1.62–1.89). Furthermore, significant correlates of lower risk of depression included sleep duration 6–8 h/day (OR = 0.45, 95% CI: 0.37–0.53) and >8 h/day (OR = 0.32, 95% CI: 0.27–0.38), exercise durations of 30–60 min/day (OR = 0.64, 95% CI: 0.60–0.70) and ≥60 min/day (OR = 0.67, 95% CI: 0.59–0.77), study duration 4–8 h/day (OR = 0.87, 95% CI: 0.78–0.98), having higher than typical study efficiency (OR = 0.64, 95% CI: 0.56–0.74), participating in distant learning (OR = 0.68, 95% CI: 0.56–0.81), and living in provinces with number of confirmed cases 1000–9999 (OR = 0.47, 95% CI: 0.24–0.92) (Table 3).

**Table 3:** Independent correlates of depression and anxiety of the whole sample (*n* = 13,440).

Variables	Depression				Anxiety			
	*P value*	*OR*	95% CI for OR		*P value*	*OR*	95% CI for OR	
			Lower	Upper			Lower	Upper
Second survey completion	**<0.001**	1.47	1.34	1.61	**<0.001**	1.64	1.48	1.81
Female	**<0.001**	1.21	1.12	1.31	**<0.001**	1.23	1.13	1.34
Senior secondary school	**<0.001**	1.41	1.29	1.54	**<0.001**	1.27	1.15	1.41
School type								
Provincial key schools	Ref				Ref			
Municipal key schools	0.551	0.95	0.80	1.13	0.064	0.84	0.69	1.01
Ordinary schools	0.336	1.08	0.92	1.26	**0.003**	0.78	0.66	0.92
Vocational schools	**0.015**	1.25	1.04	1.51	0.327	0.90	0.74	1.11
Major class	0.050	0.92	0.84	1.00	0.265	1.06	0.96	1.17
Sleep duration/day								
<6 h	Ref				Ref			
6–8 h	**<0.001**	0.45	0.37	0.53	**<0.001**	0.47	0.40	0.55
>8 h	**<0.001**	0.32	0.27	0.38	**<0.001**	0.34	0.28	0.40
Study duration/day								
<4 h	Ref				Ref			
4–8 h	**0.023**	0.87	0.78	0.98	0.098	0.89	0.78	1.02
>8 h	0.758	1.02	0.90	1.16	0.096	1.13	0.98	1.31
Exercise duration/day								
<30 min	Ref				Ref			
30–60 min	**<0.001**	0.64	0.60	0.70	**<0.001**	0.73	0.67	0.80
>60 min	**<0.001**	0.67	0.59	0.77	**0.025**	0.84	0.73	0.98
Change of study efficiency								
Lower than before	Ref				Ref			
About the same	**<0.001**	0.65	0.60	0.71	**<0.001**	0.72	0.65	0.79
Higher than before	**<0.001**	0.64	0.56	0.74	**0.014**	0.81	0.69	0.96
No. of COVID-19 cases at provincial level								
10–99	Ref				Ref			
100–999	0.159	0.62	0.32	1.21	**0.008**	0.41	0.22	0.80
1000–9999	**0.028**	0.47	0.24	0.92	**0.001**	0.31	0.16	0.60
>10,000	0.135	0.59	0.30	1.18	**0.013**	0.42	0.21	0.83
Participating in distance learning	**<0.001**	0.68	0.56	0.81	0.637	0.95	0.77	1.18
Concerned about entering a higher grade	**<0.001**	1.75	1.62	1.89	**<0.001**	1.74	1.59	1.91

Due to Collinearity between age and grade, age was not entered in the multivariable logistic regression analysis.

Bolded values: *P* < 0.05.

*CI* confidential Interval, *OR* odds ratio.

Similarly, increased risk of anxiety was related to completing the second survey (OR = 1.64, 95% CI: 1.48–1.81), female gender (OR = 1.23, 95% CI: 1.13–1.34), senior secondary school (OR = 1.27, 95% CI: 1.15–1.41), and being concerned about entering a higher grade (OR = 1.74, 95% CI: 1.59–1.91). Lower risk of anxiety was independently associated with studying in ordinary schools (OR = 0.78, 95% CI: 0.66–0.92), sleep duration 6–8 h/day (OR = 0.47, 95% CI: 0.40–0.55) and >8 h/day (OR = 0.34, 95% CI: 0.28–0.40), exercise durations of 30–60 min/day (OR = 0.73, 95% CI: 0.67–0.80) and ≥60 min/day (OR = 0.84, 95% CI: 0.73–0.98), having higher than typical study efficiency (OR = 0.81, 95% CI: 0.69–0.96), and living in provinces with number of confirmed cases >100 (OR = 0.41, 95% CI: 0.22–0.80) (Table 3).

## Discussion

This was the first study to compare rates of depression and anxiety among adolescents during versus after the initial COVID-19 outbreak. Compared with the corresponding figures during the initial COVID-19 outbreak in China, the prevalence of depression and anxiety significantly increased after the initial COVID-19 outbreak had remitted.

Compared to the prevalence of depression (24.3%; 95% CI: 21.3–27.6%)^30^ and anxiety (14.1%; 95% CI: 13.2–15.0%)^31^ among Chinese adolescents prior to the COVID-19 outbreak, both depression (36.6%; 95% CI: 35.6–37.6%) and anxiety (19%; 95% CI: 18.2–19.8%) were more common during the COVID-19 outbreak. These increases could be due to unpleasant experiences brought by the COVID-19 outbreak such as fear of infection, limited public services, frustration, loneliness or lack of interpersonal contact with peers, and limited personal space at home.

Significant increases in prevalence of depression and anxiety among adolescents in the second survey may be attributed to the several factors. First, although timely online mental health education and services have been established during the COVID-19 outbreak in China^6,32^, their content is not designed for special populations such as adolescents. On a related note, most secondary school students were highly occupied with heavy study burdens that might interfere with involvement and benefits of these mental health services. Second, COVID-19 was well controlled in China during the second survey but most students suffering from depressive and/or anxiety symptoms during the first survey did not receive timely effective treatments due to inaccessible mental health services in many areas; as such, symptoms may not have remitted. Third, due to school closures and social distancing, adolescents may have experienced losses of close peer relationships, which could increase risk for depression and anxiety^18^. Furthermore, lack of outdoor exercise^33,34^ and prolonged use of the Internet and smart phones at home^35,36^ could increase risk for these disturbances. Fourth, in the second survey administration (i.e., after the initial COVID-19 outbreak), 88.3% of students still had not returned to school, although most of their parents had returned to work according to the relevant regulations in China. Lack of clarity about returning to school during this semester as well as lack of care and supervision from parents could have contributed to further increases in the risk for mental health problems. Finally, final examinations that are traditionally held in May–June were approaching yet face-to-face teaching could not be offered to many students. This combination may have increased the likelihood of mental health problems.

Consistent with previous findings^16,37^, female adolescents and those in senior secondary school were more likely to have anxiety and depression. We also found students who reported daily study durations of more than 8 h and concerns about entering a higher grade were more likely to report depression and anxiety. Long hours of study are often associated with heavy homework demands, insufficient sleep, and overuse of computers, all of which could have a negative impact on adolescent mental health^20,38,39^. In China, secondary school students in higher grades often face higher academic stress levels^40,41^ because they need to prepare for college entrance examinations that have significant implications for future education and occupation opportunities^41,42^. Because many students could not receive traditional teaching due to the COVID-19 outbreak, they may have been more likely to suffer from depression and anxiety.

In this study, adolescents whose sleep durations were more than 6 h/day, whose exercise durations were more than 30 min/day, and whose study efficiency was higher than the level perceived before the COVID-19 outbreak were at significantly lower risk of depression and anxiety, results that echo previous findings^43,44^. Additionally, adolescents enrolled in ordinary schools were less likely to have anxiety compared to those in key schools where higher levels of academic stress are expected due to higher teacher and parent performance expectations and increased peer competition^40,45,46^. Adolescents participating in distance learning had a lower risk of depression, which may be attributed, in part, to more frequent communication and support from teachers and classmates^47^, online communication experiences that can reduce negative feelings of adolescents^48,49^. Finally, to control the spread of infection, provinces with more confirmed COVID-19 cases usually have more strict quarantine measures as well as timely mental health preventive measures and education. Aside from improving residents’ knowledge about COVID-19, these resources could enhance their sense of control and confidence in battling the COVID-19 outbreak, in turn, reducing the negative impact of higher COVID-19 rates on their mental health^5,20^. In line with these contentions, this study confirmed that adolescents living in provinces with more than 100 confirmed COVID-19 cases had a lower risk of anxiety.

Strengths of this study included its two-round survey and the large sample size. Several limitations should also be noted. First, some potentially important correlates of depression or anxiety such as social support and physical health, could not be examined for logistical reasons (e.g., absence of resources to perform physical exams). Second, causal relationships between mental health problems and related variables could not be examined due to the cross-sectional design and absence of experimental manipulations. Third, standardized diagnostic instruments such as the Structured Clinical Interview for the DSM-IV (SCID) could not be administered due to safety requirements associated with minimizing face-to-face contacts during the outbreak. Finally, because students with pre-existing major psychiatric disorders were excluded, findings may not generalize to this group.

In conclusion, depression and anxiety were common among adolescents in China during the initial COVID-19 outbreak and their prevalence increased significantly ~6 weeks later even though the outbreak was more under control. Although psychological interventions such as hotlines and online psychological counseling services, had been provided for the general public, students may have been less able to directly benefit from the generic content of such resources. Therefore, considering the negative impact of depression and anxiety on daily life and health outcomes, timely screening and appropriate interventions, such as online psychological counseling tailored for concerns specific to adolescents, are urgently needed to reduce the likelihood of emotional disturbances among adolescents during and after the initial COVID-19 outbreaks.
